# Universal buffers for use in biochemistry and biophysical experiments

**DOI:** 10.3934/biophy.2015.3.336

**Published:** 2015-08-14

**Authors:** Dewey Brooke, Navid Movahed, Brian Bothner

**Affiliations:** Department of Chemistry and Biochemistry, Montana State University, Bozeman MT 59717, USA

**Keywords:** buffer, protein, pH, enzyme assay, structure, allostery

## Abstract

The use of buffers that mimic biological solutions is a foundation of biochemical and biophysical studies. However, buffering agents have both specific and nonspecific interactions with proteins. Buffer molecules can induce changes in conformational equilibria, dynamic behavior, and catalytic properties merely by their presence in solution. This effect is of concern because many of the standard experiments used to investigate protein structure and function involve changing solution conditions such as pH and/or temperature. In experiments in which pH is varied, it is common practice to switch buffering agents so that the pH is within the working range of the weak acid and conjugate base. If multiple buffers are used, it is not always possible to decouple buffer induced change from pH or temperature induced change. We have developed a series of mixed biological buffers for protein analysis that can be used across a broad pH range, are compatible with biologically relevant metal ions, and avoid complications that may arise from changing the small molecule composition of buffers when pH is used as an experimental variable.

## 1. Introduction

The use of buffers that mimic biological solutions is a foundation of biochemical studies. One of the most common buffering agents is phosphate-buffered saline (PBS) which was formulated to match the ionic strength and pH of mammalian cells. While it works very well in many experiments, there are troublesome properties including a complex formation with divalent cations such as calcium (Ca^2+^) leading to precipitation and interaction with proteins [1–3]. However, PBS is on a short list of buffers for which the acid disassociation constant (K_a_) does not have a strong temperature dependence. A quick look at the chemical shelves in most biochemistry laboratories will reveal a large number of buffers. This is because the ability to obtain biochemical or biophysical data depends on finding solution conditions in which proteins are soluble, stable, and retain activity. Herein we provide a few of poignant examples of buffer effects, in addition to those for PBS above, and then describe a simple solution that is applicable to a wide range of experimental approaches.

Enzymologists have been acutely aware of the influence a buffer can have on kinetic parameters for nearly half a century, realizing that making comparisons between conditions was problematic [4,5]. More recently structural biologists have come to appreciate the influence of solvent molecules on protein structure and dynamics. A study of selenocysteine synthase using X-ray crystallography reported that phosphate buffer caused a previously unstructured loop to fold into a phosphate-binding domain [6]. A more dramatic report by, Long *et al*. was the first to focus on the magnitude of buffer effects on protein dynamics [7]. Using NMR, they demonstrated that a weak interaction between human liver fatty acid binding protein (hLFABP) and the buffering agent 2-(N-Morpholino)ethanesulfonic acid (MES) caused a significant change in the conformational dynamics at the microsecond to millisecond timescale without affecting structure [7]. This study went on to show that Bis-Tris, also altered the conformational dynamics of hLFABP, yielding a different ensemble of conformations. Phosphate and calcium are common components of biological buffers and are important protein ligands. A change in their concentration can have a major impact on protein structure and function [2,8,9]. These examples highlight the significant influence solution composition can have on the properties of proteins and emphasize the attention to detail that is required in structure-function studies.

Proteins that are secreted from a cell or localized to a subcellular organelle often experience a range of pHs. The importance of *in vivo* pH changes is well appreciated and methods for monitoring this to better understand processes have been developed [10]. Therefore, conducting biophysical characterizations and activity assays at different pHs, can provide important insights to biological mechanism and role. To maintain buffering capacity in an assay, it is common for buffers to be changed across a pH gradient. In light of the examples presented above, this raises serious questions about how to separate buffer effects from pH effects. The standard method of basing buffer selection on the useful pH becomes an issue with biomolecules that have specific or nonspecific interactions with solute molecules. Studies of viral entry via endocytosis, highlight this issue [11,12,13]. For example, to understand the mechanisms that Adeno-associated viruses use to escape from endosomes, *in vitro* experiments must use a range of pHs, just as occurs during endosome acidification *in vivo* [14]. In fact, the whole life cycle of a virus is a thermodynamic balancing act between assembly and disassembly, a process that is regulated by ionic strength, cation concentration, and pH [15]. Even minor buffer effects can have a major impact on the solution-phase behavior.

A single buffering solution capable of performing across relevant wide pH range would greatly simplify interpretation of protein activity data and enhance biological understanding. A few universal-buffers with a broad working pH range (2–12) have been described, however, they are composed of compounds that interact unfavorably with proteins, or chelate metal-ions that are required for protein structure and function [16]. The goal of this study was to formulate a set of buffers with general suitability for biochemical research that span a wide pH range without the to alter composition. Other factors such as compatibility with common biological divalent cations and the temperature dependence of pKa are also addressed. To this end, three different biocompatible universal buffers with working ranges of 3–9 are presented.

## 2. Methods

Sodium Acetate Trihydrate, 4-(2-hydroxyethyl)-1-piperazineethanesulfonic acid (HEPES), and Tris(hydroxymethyl)aminomethane (Tris) were purchased from Thermo Fisher Scientific Inc. MES sodium salt was purchased from Sigma as an anhydrate. Bis-Tris (2,2-Bis(hydroxymethyl)-2,2’,2”-nitrilotriethanol) was purchased from Acros Organics, and Tricine (N-Tris[hydroxymethyl]methylglycine was purchased from BioRad.

Each universal buffer is an equimolar mixture of three reagents with each performing as a buffer when the pH is near its pKa. Universal buffers were prepared by adding appropriate amounts of individual dried buffer to distilled water. To standardize concentration for each universal buffer used in the titration and temperature experiments, the sum concentration of reagents was 60mM for each formulation. For the titrations, pH of the individual buffer and universal buffers was set to pH 11 using 10M sodium hydroxide and then brought to final concentration by addition of water. Titrations from high to low pH were conducted by step-wise addition of 5M hydrochloric acid followed by vigorous mixing. pH was measured using a standard pH electrode (Thermo Fisher Scientific Dj Glass AG/AGCL Ph Electrode with a waterproof BNC).

## 3. Results

**Figure 1.**
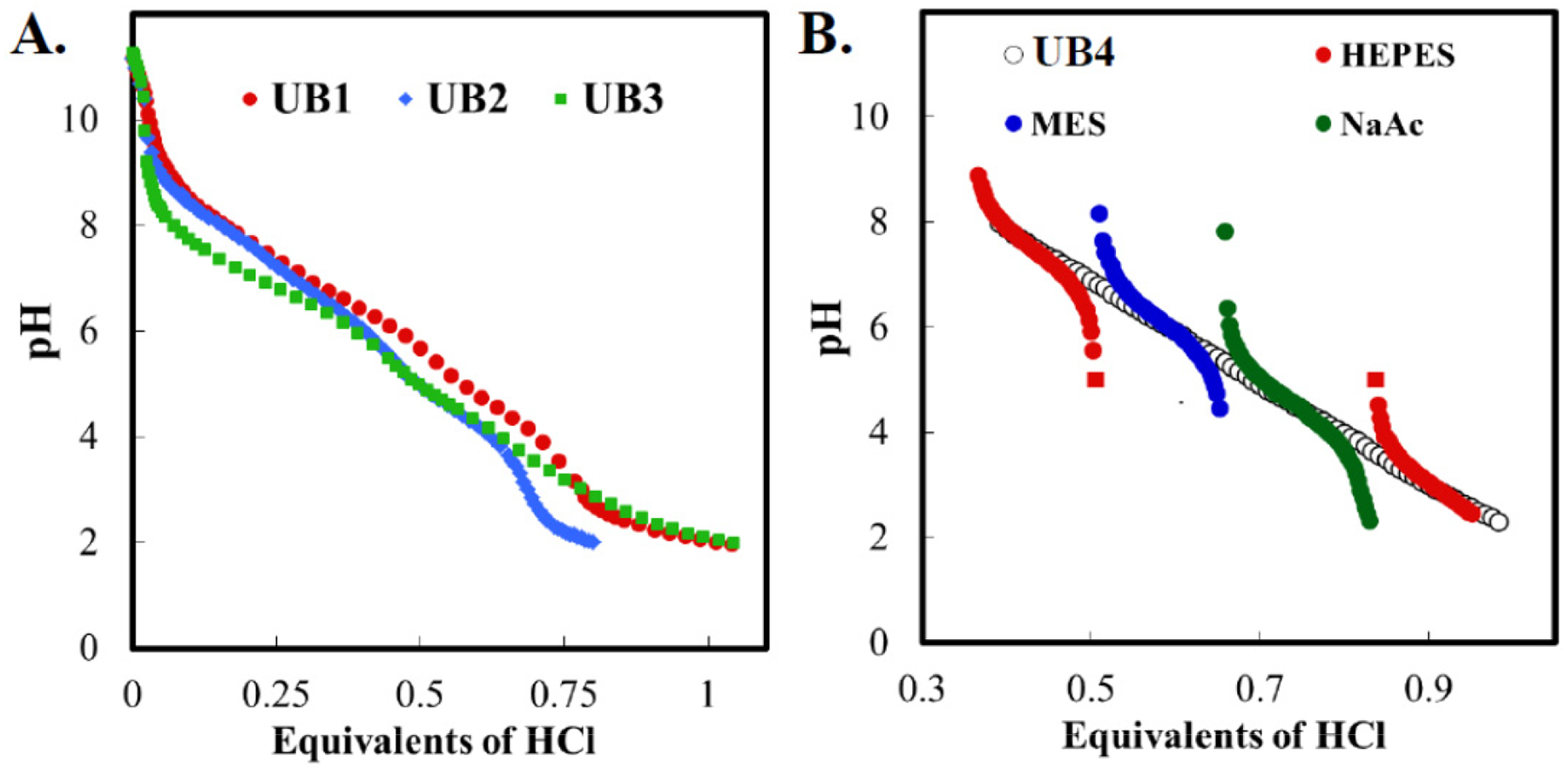
Titration curves of universal buffers A. Titration curves for three different universal buffers. UB1 (Tricine, Bis-Tris, sodium acetate), UB2 (Tris-HCl, Bis-Tris, sodium acetate), UB3 (HEPES, Bis-Tris, sodium acetate). B. Titration curves of separate and combined components of UB4 (HEPES, MES, and sodium acetate) with the titration curve of its components. Individual curves for HEPES, MES, and NaAc show buffering range of specific weak acids. HEPES is a diprotic acid and has two relevant pKa’s. When combined into a universal buffer, a linear response to the addition of acid is observed across the entire tested range.

The ability of a solution to resist pH changes during addition or removal of protons is due to the presence of a weak acid and the conjugate base. Because protonated and deprotonated species must be present in appreciable quantities, any donor/acceptor only offers significant buffering capacity when the solution pH is within ~1 unit of the pKa [17]. Our first interest was to create a solution that could buffer across the pH range of 2–9. Good’s buffers for biological research [18] was a starting point for selecting compounds with pKa’s close to 7, 5, and 3. The first universal buffer (UB) tested was composed of Tricine (pKa 8.05), Bis-Tris (pKa 6.46), and sodium acetate (pKa 4.76). The titration curve of UB1 was nearly linear from pH 3.5–9 (Figure 1A). A second composite buffer was produced to reduce interaction with Ca^2+^ and Mg^2+^, divalent cations that are present at mM concentrations in cellular cytoplasm and biological fluids [19]. Tricine affinity for divalent cations (see Table 1) makes it incompatible with solutions containing low mM concentrations of Ca^2+^ and Mg^2+^. Tris (pKa 8.01) and HEPES (pKa 7.55) were tested as substitutes for Tricine in UB. A new Tris UB (UB2) with a working range of pH 3.5–9 and HEPES UB (UB3) for pH 2–8 were tested. (Figure 1A). A fourth UB, composed of HEPES, MES, and sodium acetate (UB4) had a linear titration curve from pH2–8 (Figure 1B). Titration curves of the individual components are shown for comparison. It is important to note that the UB2, UB3, and UB4 are not devoid of interactions with metal ions. For example, Tris forms complexes with Cu(II), Ni(II), Zn(II), Ag(I), and Co(II) [20]. However, these interactions are negligible at all but the most extreme biologically relevant.

Biological, biochemical, and biophysical experiments are often conducted at different temperatures. This may be to study lethality of a temperature sensitive mutant, determine the thermodynamic properties of a protein, or characterize the microenvironment of a fluorescent probe [22,23]. The actual solution pH in these experiments becomes a concern since increasing temperature can change a buffer’s pKa due to changes in chemical potential. For example, a Tris-HCl buffer will change ~2 pH units during a protein thermal denaturation experiment which goes from 298–373K. Such a large pH change significantly complicates data interpretation. The temperature dependence of each UB was measured in order to access suitability for use in experiments in which temperature is varied. At pH7, UB3 and UB4 had a temperature dependence of −0.014 pKa/°C. At the same pH UB1 changed −0.015 pKa/°C (Table 1). UB2 had the largest temperature dependence, −0.020 pKa/°C due to the presence of Tris. In all cases, the observed temperature dependence of pKa for the UBs was equal to or less than that of the components alone.

## 4. Conclusion

We have introduced a series of biological buffers suitable for use across a broad pH range. Universal buffers have been presented before [16], but they were not suitable for biological research. Three of the four UB have negligible metal-binding affinity making them suitable for studying enzyme reactions, protein structure/dynamics, and cell signaling that require divalent cations such as Mg^2+^, Ca^2+^, and Cu^2+^. We have also measured the temperature dependence of the UBs to allow selection of a buffer suitable for experiments in which temperature changes occur. The increasing precision of biochemical research in general and studies of protein dynamics, drug screening, and membrane channel activity specifically will benefit from use of a single buffer solution throughout an experiment, or can serve as a control against solute driven changes in protein behavior.

## Figures and Tables

**Table 1.** Physical Properties of buffers.

Buffer	pKa at 25°C	dpKa/°C at pH 7.0	MW	Metal Binding
Bis-Tris	6.46	N/A	209.24	Negligible^1^
HEPES	7.55	−0.014	238.3	Negligible^1^
MES	6.15	−0.011	195.2	Negligible^1^
NaAc	4.76	Negligible^1^	82.03	Negligible^1^
Tricine	8.05	−0.021	179.2	Ca^2+^, Mg^2+^, Mn^2+^, Cu^2+^
Tris	8.06	−0.028	121.14	Negligible^1^
	**Buffer Range**			
UB1^a^	3.0–9.0	−0.015		Ca^2+^, Mg^2+^, Mn^2+^, Cu^2+^
UB2^b^	3.5–9.2	−0.020		Negligible^1^
UB3^c^	2.0–8.2	−0.012		Negligible^1^
UB4^c^	2.0–8.2	−0.012		Negligible^1^

a. 20mM Tricine, 20mM Bis-Tris, and 20mM sodium acetate,

b. 20mM Tris-HCl, 20mM Bis-Tris, and 20mM sodium acetate.

c. 20mM HEPES, 20mM Bis-Tris, and 20mM sodium acetate.

d. 20mM HEPES, 20mM MES, and 20mM sodium acetate

1. Negligible in standard biophysical assays [12, 14.^15^]

## Abbreviations

PBS: phosphate-buffered saline
MES: 2-(N-Morpholino)ethanesulfonic acid
hLFABP: human liver fatty acid binding protein
NMR: nuclear magnetic resonance
Tris-HCl: 2-Amino-2-hydroxymethyl-propane-1,3-diol hydrochloric acid
HEPES: 4-(2-hydroxyethyl)-1-piperazineethanesulfonic acid
Bis-Tris: 2-Bis(2-hydroxyethyl)amino-2-(hydroxymethyl)-1,3-propanediol
Tricine: N-(2-Hydroxy-1,1-bis(hydroxymethyl)ethyl)glycine
UB: universal buffer

## References

[1] Pascal SM, Yamazaki T, Singer AU, (1995) Structural and dynamic characterization of the phosphotyrosine binding region of a Src homology 2 domain--phosphopeptide complex by NMR relaxation, proton exchange, and chemical shift approaches. Biochemistry 34:11353–11362. 7547863 10.1021/bi00036a008

[2] Zhang M, Zhou M, van Etten RL, (1997) Crystal structure of bovine low molecular weight phosphotyrosyl phosphatase complexed with the transition state analog vanadate. Biochemistry 36:15–23. 8993313 10.1021/bi961804n

[3] Katayama DS, Nayar R, Chou DK, (2006) Effect of buffer species on the thermally induced aggregation of interferon-tau. J Pharm Sci 95:1212–1226. 16637050 10.1002/jps.20471

[4] Hausamen TU, Helger R, Rick W, (1967) Optimal conditions for determination of serum alkaline phosphatase by a new kinetic method. Clin Chim Acta 15: 241–245.

[5] McComb RB, Bowers GN (1972) Study of optimum buffer conditions for measuring alkaline-phosphatase activity in human serum. Clin Chem 18: 97–103. 5008530

[6] Ganichkin OM, Xu XM, Carlson BA, (2008) Structure and catalytic mechanism of eukaryotic selenocysteine synthase. J Biol Chem 283: 5849–5865. 18093968 10.1074/jbc.M709342200

[7] Long D, Yang D (2009) Buffer Interference with Protein Dynamics: A Case Study on Human Liver Fatty Acid Binding Protein. Biophys J 96 1482–1488. 19217864 10.1016/j.bpj.2008.10.049 PMC2717250

[8] Sopkova J, Renouard M, Lewit-Bentley A (1993) The crystal structure of a new high-calcium form of annexin V. J Mol Biol 234: 816–825. 8254674 10.1006/jmbi.1993.1627

[9] Kumar S, Sharma P, Arora K, (2014) Calcium binding to beta-2-microglobulin at physiological pH drives the occurrence of conformational changes which cause the protein to precipitate into amorphous forms that subsequently transform into amyloid aggregates. PLoS One 9: e95725. 24755626 10.1371/journal.pone.0095725 PMC3995793

[10] Schmitt FJ, Thaa B, Junghans C, (2014) eGFP-pHsens as a highly sensitive fluorophore for cellular pH determination by fluorescence lifetime imaging microscopy (FLIM). Biochim Biophys Acta 1837: 1581–1593. 24742974 10.1016/j.bbabio.2014.04.003

[11] Bothner B, Schneemann A, Marshall D, (1999) Crystallographically identical virus capsids display different properties in solution. Nat Struct Bio 6: 114–116. 10048920 10.1038/5799

[12] Bothner B, Taylor D, Jun B, (2005) Maturation of a tetravirus capsid alters the dynamic properties and creates a metastable complex. Virology 339: 145–145. 10.1016/j.virol.2005.01.017 15749119

[13] Nam HJ, Gurda BL, McKenna R, (2011) Structural Studies of Adeno-Associated Virus Serotype 8 Capsid Transitions Associated with Endosomal Trafficking. J Virol 85: 11791–11799. 21900159 10.1128/JVI.05305-11 PMC3209291

[14] Bartlett JS, Wilcher R, Samulski RJ (2000) Infectious entry pathway of adeno-associated virus and adeno-associated virus vectors. J Virol 74: 2777–2785. 10684294 10.1128/jvi.74.6.2777-2785.2000 PMC111768

[15] Roos WH, Bruinsma R, Wuite GJL (2010) Physical virology. Nat Phys 6: 733–743.

[16] Ellis DA (1961) New universal buffer system. Nature 191: 1099–1100. 13726462 10.1038/1911099a0

[17] Henderson Y (1908) Acapnia and Shock: Carbon-dioxid as a factor in the regulation of the heart-rate. Am J Physiol 21: 126–156.

[18] Good NE, Winget GD, Winter W, (1966) Hydrogen ion buffers for biological research. Biochemistry 5: 467–472. 5942950 10.1021/bi00866a011

[19] Garrett RH, Charles M, Grisham CM (2012). Biochemistry 5th ed. Cengage Learning.

[20] Sokolowska M, Bal J (2005) Cu(II) complexation by “non-coordinating” N-2-hydroxyethylpiperazine-N′−2-ethanesulfonic acid (HEPES buffer) J Inorg Biochem 99: 1653–1660. 15993944 10.1016/j.jinorgbio.2005.05.007

[21] Taha M (2011) Complex Equilibria in Aqueous Solutions of Chromium(III) with Some Biological pH Buffers. J Chem Eng Data 56: 3541–3551.

[22] Shnyrov VL, SanchezRuiz JM, Boiko BN, (1997) Applications of scanning microcalorimetry in biophysics and biochemistry. Thermochim Acta 302: 165–180.

[23] Lakowicz J (2006) Principles of Fluorescence Spectroscopy. New York: Springer.

